# A lightweight cerebrospinal fluid biomarker-based model for first-diagnosis prediction of Parkinson’s disease: model development, external validation, and local deployment

**DOI:** 10.3389/fnagi.2025.1723169

**Published:** 2025-12-16

**Authors:** Xinchao Hu, Yu Liu, Yuan Cao, Chunli Wei, Kun Liu, Jing-Hua Yang

**Affiliations:** 1 Clinical Systems Biology Center, The First Affiliated Hospital of Zhengzhou University, Zhengzhou, China; 2 Reproductive Medicine Center, The First Affiliated Hospital of Zhengzhou University, Zhengzhou, China; 3 Department of Neurology, The First Affiliated Hospital of Zhengzhou University, Zhengzhou, China

**Keywords:** Parkinson’s disease, cerebrospinal fluid biomarkers, machine learning, early diagnosis, local model deployment

## Abstract

**Background:**

Despite substantial progress in biomarker research, Parkinson’s disease (PD) still lacks widely validated, easily deployable diagnostic tests for reliable early-stage detection, particularly in resource-limited circumstances.

**Objective:**

This study aimed to develop and externally validate a lightweight machine learning model for the first-diagnosis prediction of PD using baseline cerebrospinal fluid (CSF) biomarkers from the Parkinson’s Progression Markers Initiative (PPMI).

**Methods:**

Baseline CSF data from 665 participants (PD = 415, controls = 190, SWEDD = 60) were used. Five machine learning classifiers—L2-regularized logistic regression (L2-LR), random forest (RF), histogram-based gradient boosting (HistGB), support vector machine with RBF kernel (SVM-RBF), and multilayer perceptron (MLP)—were trained and compared. Feature selection focused on five core CSF biomarkers (Aβ42, α-synuclein, total tau, phosphorylated tau181 and hemoglobin). Model performance was evaluated using AUC, PR-AUC, and Brier scores, followed by isotonic calibration and independent validation using the University of Pennsylvania dataset.

**Results:**

A lightweight, biomarker-based RF model effectively distinguishes first-diagnosis PD cases using limited baseline CSF indicators. Its offline Streamlit deployment offers a practical tool for resource-limited settings, bridging the gap between computational prediction and real-world neurological diagnosis.

## Introduction

Parkinson’s disease (PD) is the second most prevalent neurodegenerative disorder globally, affecting an estimated 1%–2% of individuals over the age of 65 (Ben-Shlomo et al., 2024). It is clinically characterized by progressive motor impairments—such as bradykinesia, rigidity, and tremor—as well as a diverse range of non-motor symptoms including cognitive decline, sleep disturbances, and autonomic dysfunction (Bloem et al., 2021). Due to its heterogeneous presentation and long prodromal phase, early and accurate diagnosis remains a major clinical challenge. Autopsy-based and longitudinal clinicopathological studies have consistently shown that a substantial proportion of patients initially labeled as PD are later reclassified, indicating that early diagnostic accuracy can be as low as 75% even in specialized centers (Räty et al., 2025). This persistent diagnostic uncertainty highlights an important unmet clinical need. Nonetheless, timely identification is critical for optimizing therapeutic interventions, facilitating patient stratification, and enabling enrollment in disease-modifying clinical trials (Bloem et al., 2021; Marsili et al., 2018).

Over the past two decades, cerebrospinal fluid (CSF) biomarkers have emerged as key tools for dissecting disease heterogeneity and cognitive trajectories in PD. α-Synuclein (α-syn), the principal constituent of Lewy bodies, is widely regarded as a pathophysiologically grounded, PD-related CSF biomarker. However, systematic reviews and meta-analyses indicate that CSF total, oligomeric, and phosphorylated α-syn are, on average, reduced in PD compared with controls. At the same time, substantial between-study heterogeneity—driven by differences in assay platforms, pre-analytical handling, and blood contamination—and partial overlap with non-PD profiles constrain the utility of CSF α-syn as a stand-alone diagnostic test (Eusebi et al., 2017; Irwin et al., 2020). It’s worth noting that beyond α-syn, total tau (t-tau), threonine 181–phosphorylated tau (p-tau181), and β-amyloid 42 (Aβ42)—canonical Alzheimer-type markers—also show systematic alterations in PD cohorts: reduced Aβ42 and/or elevated tau levels are tightly associated with cognitive decline, coexisting Alzheimer pathology, and accelerated clinical progression (Mantovani et al., 2024; Montine et al., 2010; Siderowf et al., 2010). Importantly, these measures are increasingly shifting from being used solely to “rule out Alzheimer’s disease” toward serving as integral components of risk stratification and prognostic staging within the PD population.

Given the modest and inconsistent group-level differences reported for individual CSF biomarkers, a single-marker strategy is unlikely to achieve clinically meaningful discrimination (Katayama et al., 2020; Yang et al., 2023; Zarkali et al., 2024). This has prompted a shift toward data-driven approaches, particularly machine learning (ML), to integrate small panels of CSF measures and enhance subject-level predictive performance (Chen et al., 2024; Luo et al., 2025; Tsukita et al., 2023). ML algorithms are well suited to capture complex, nonlinear relationships among biological variables and are therefore attractive candidates for biomarker-based precision diagnostics in neurodegenerative disease. Yet most existing ML frameworks rely on computationally intensive architectures, cloud-based infrastructure, or proprietary software and remain confined to research settings, with limited translation into point-of-care tools. This translational gap is especially pronounced in resource-limited environments, where constrained computational capacity and software accessibility further hinder real-world deployment (Aborode et al., 2025; Ahmed et al., 2023; Voigtlaender et al., 2024; Yousefi et al., 2024).

Importantly, because CSF sampling is invasive and resource-intensive, any CSF-based diagnostic strategy must demonstrate clinical value while remaining simple, transparent, and easy to deploy. Lightweight ML models that operate offline on standard hospital computers and use only a minimal set of widely available biomarkers are therefore particularly attractive for implementation. In this context, we set out to (i) assess the discriminative potential of five key CSF biomarkers, (ii) develop and compare multiple supervised ML classifiers for individualized PD prediction, and (iii) implement a lightweight, locally deployable diagnostic tool capable of real-time, offline inference from minimal biomarker input. By addressing both biological and practical constraints, our work aims to advance the integration of fluid biomarkers into scalable, interpretable, and clinically accessible decision-support tools for aging-related neurodegenerative disease.

## Materials and methods

### Data source and study cohort

This study used fully de-identified human data from the Parkinson’s Progression Markers Initiative (PPMI) cohort, accessed via the Accelerating Medicines Partnership–Parkinson’s disease (AMP-PD) knowledge platform. This study used fully de-identified baseline (“first-visit”) data from the *de novo* PPMI cohort. The analytic sample comprised 918 baseline participants: 656 individuals with newly diagnosed, drug-naïve PD, 202 healthy controls, and 60 subjects with scans without evidence of dopaminergic deficit (SWEDD). Enrollment criteria for these first-visit *de novo* PD, SWEDD, and control cohorts followed the original PPMI protocol. *De novo* PD participants were required to be ≥ 30 years of age, have idiopathic PD of ≤ 2 years’ duration at screening, Hoehn–Yahr stage < 3, and either (i) at least two of rest tremor, bradykinesia, or rigidity (with rest tremor or bradykinesia mandatory) or (ii) asymmetric rest tremor or asymmetric bradykinesia alone; they were drug-naïve for PD medications or had received them for ≤ 60 days in total and not within 60 days of the baseline visit. Individuals with similar first-visit clinical features but normal DAT/VMAT-2 imaging were enrolled in the SWEDD cohort. Healthy controls were ≥ 30 years of age, had no clinically significant neurological disorder or first-degree relative with PD, and scored > 26 on the Montreal Cognitive Assessment.

### Ethical approval

The PPMI study was approved by the institutional review boards of all participating sites, and written informed consent was obtained from all participants. Because only de-identified data were analyzed, the present study was exempt from additional ethics review under local institutional regulations.

### Biomarker selection and data preprocessing

We initially treated all available CSF analytes as candidate predictors and used a data-driven feature-selection pipeline. Biomarkers measured in the majority of participants were retained, and exploratory classification models including these candidates were trained to assess cross-validated area under the curve (AUC) and model-based importance. Variables with negligible or unstable contribution to discrimination were iteratively removed, yielding a compact 5-marker panel: α-syn, Aβ42, t-tau, p-tau181, and hemoglobin.

**FIGURE 1:**
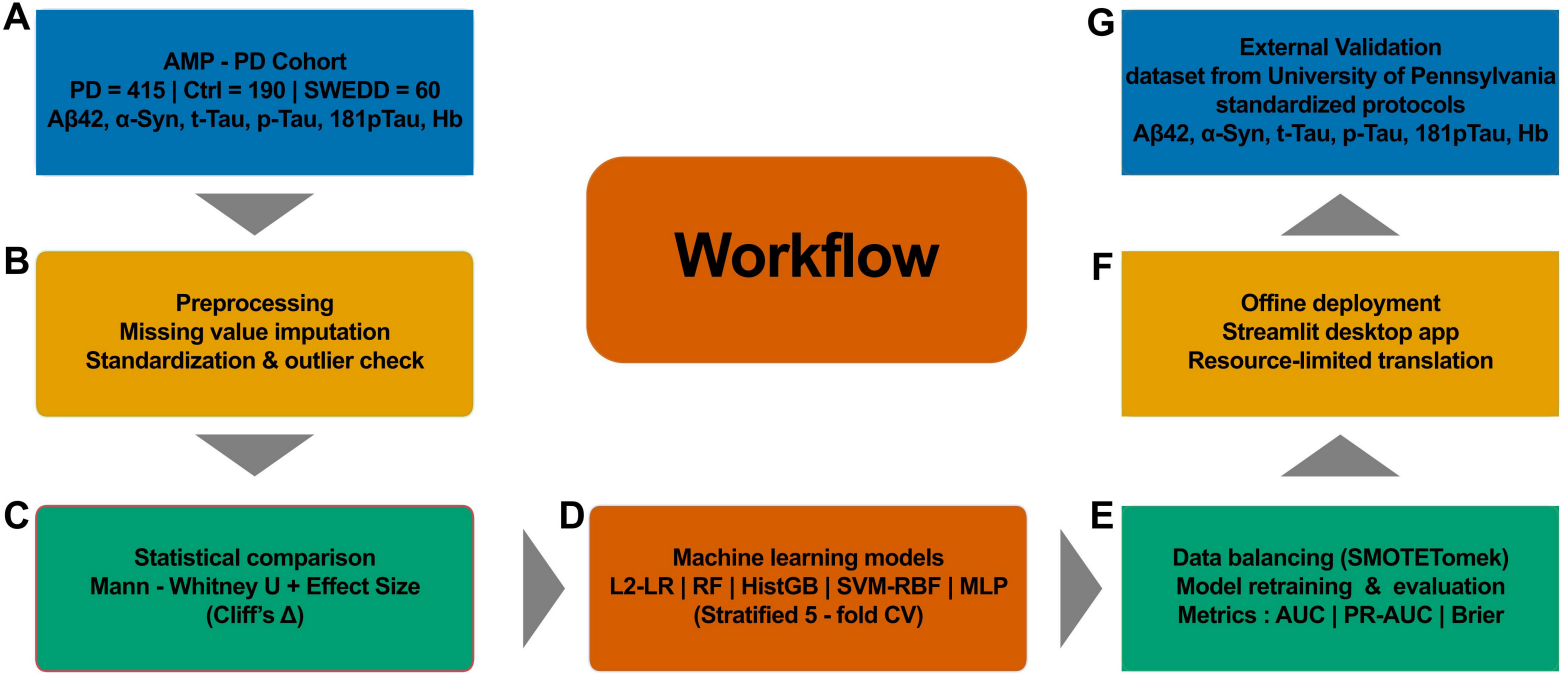
Overall workflow of the CSF biomarker-based PD prediction model. (A) AMP-PD cohort with PD, control, and SWEDD participants and six CSF biomarkers. (B) Preprocessing including missing-value imputation, standardization, and outlier screening. (C) Group comparison using Mann-Whitney U tests with effect-size estimation. (D) Training of five machine-learning models with stratified 5-fold cross-validation. (E) Data balancing using the SMOTE-Tomek method and performance evaluation based on AUC, PR-AUC, and Brier score. (F) Offline deployment through a lightweight Streamlit desktop application. (G) External validation using an independent dataset from the University of Pennsylvania with standardized biomarker protocols.

For these biomarkers, participants missing all five measures were excluded. All baseline PPMI participants with available CSF measurements for the five biomarkers and diagnostic classification were included, yielding 605 individuals: 415 patients with *de novo* PD and 190 age- and sex-matched healthy controls, plus 60 participants with scans without evidence of dopaminergic deficit (SWEDD). SWEDD participants were retained for descriptive and exploratory visualization but were excluded from model training and validation. Remaining missing values were imputed using within-group (PD vs. control) medians. Outliers were defined using the interquartile range (IQR) rule (> 1.5 × IQR beyond the first or third quartile) and winsorized to the nearest in-range value. Because α-syn, t-tau, p-tau181, and Aβ42 showed right-skewed distributions, they were log_10_-transformed, whereas hemoglobin was analyzed on its original scale. All features were then z-score normalized within the training folds only to prevent data leakage. The full preprocessing and modeling workflow is summarized in Figure 1, and a tabular summary of biomarker definitions and preprocessing steps is provided in Supplementary Table 1.

### Feature importance and model interpretability

**FIGURE 2:**
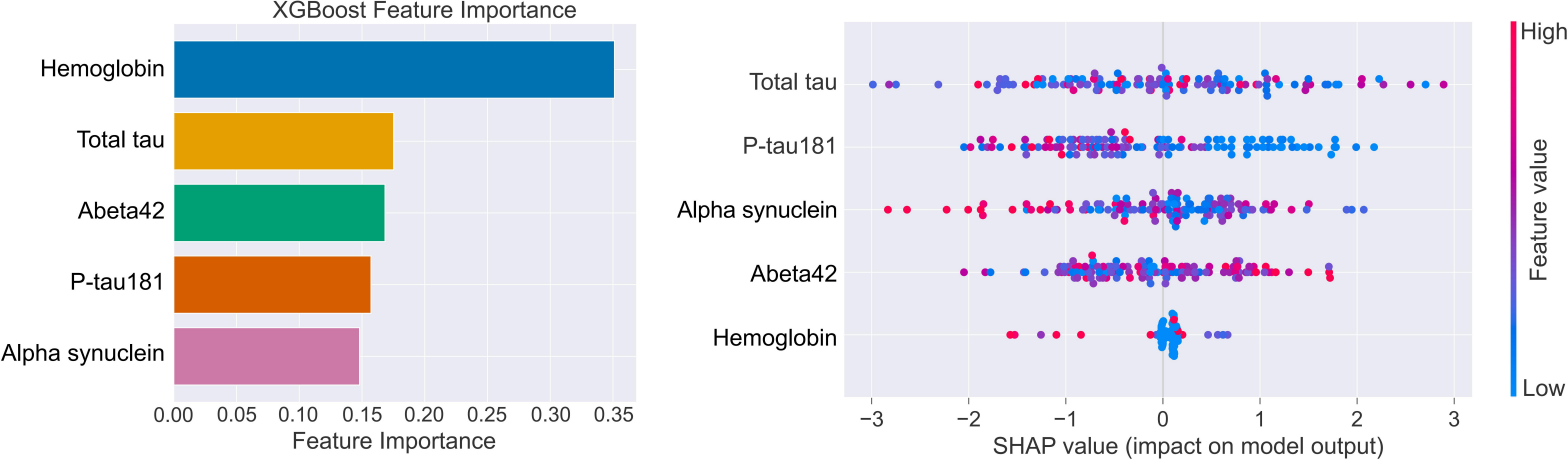
Feature importance and SHAP-based interpretability of the PPMI CSF biomarker model. In the SHAP summary plot, red points denote higher feature values and blue points denote lower feature values; the horizontal position of each point indicates the direction and magnitude of that feature’s contribution to the predicted PD probability.

For the final gradient-boosted decision tree model, global feature importance was quantified using the gain-based importance metric, defined as the average improvement in the splitting criterion attributable to each predictor across all trees in the ensemble. To obtain model-agnostic, locally valid explanations, Shapley additive explanation (SHAP) values were additionally computed for each feature and each individual. Global importance was summarized as the mean absolute SHAP value across all subjects, and the directionality and non-linear effects of each biomarker were visualized using SHAP summary and dependence plots (Figure 2). Concordance between gain-based feature ranking and SHAP-based attributions was used to assess the internal consistency of the model’s learned structure and its *post hoc* explanations.

### Statistical analysis

Because all CSF biomarkers violated normality assumptions (Shapiro–Wilk *P* < 0.05), group differences between PD and controls were assessed using the Mann–Whitney U test. Effect sizes were quantified using Cliff’s Delta with 95% confidence intervals (CI) computed via 1000-iteration bootstrapping. To control for multiple testing across the five biomarkers, false discovery rate (FDR) correction (Benjamini–Hochberg) was applied.

Multivariable logistic regression including all five biomarkers was performed to evaluate their independent associations with PD status. Age and sex were included as covariates in all logistic regression models to account for potential confounding. Odds ratios with 95% confidence intervals were reported. All analyses were conducted in Python 3.10 using SciPy 1.11 and statsmodels 0.14.

### Machine learning modeling

Five supervised ML algorithms were implemented to construct predictive models: L2-regularized logistic regression (L2-LR), random forest (RF), histogram-based gradient boosting (HistGB), support vector machine with RBF kernel (SVM-RBF), and multilayer perceptron (MLP). Model training and evaluation were conducted using five-fold stratified cross-validation. To address class imbalance, the Synthetic Minority Over-sampling Technique with Tomek Links (SMOTE–Tomek) was applied within each training fold. The following performance metrics were computed and averaged across folds: accuracy, precision, recall (sensitivity), F1-score, area under the receiver operating characteristic curve (AUC-ROC), and precision–recall (PR) performance summarized by both the area under the precision–recall curve (PR-AUC) and average precision (AP). Probabilistic calibration was assessed using the Brier score.

### Explainability and SHAP analysis

To enhance model transparency and facilitate clinical interpretability, SHAP were used to quantify both global and individual-level feature contributions. For global interpretability, TreeExplainer was applied to the final random forest model to compute the marginal contribution of each CSF biomarker across the AMP-PD cohort. These values were summarized using SHAP beeswarm plots to visualize the distribution and direction of feature effects.

### Local deployment and interface implementation

The best-performing classifier was embedded into a locally executable diagnostic application developed using Python and the Streamlit framework. The tool was designed to run entirely offline without reliance on cloud-based services, ensuring suitability for resource-limited clinical environments.

The application was deployed and tested on a macOS environment (MacBook Pro, Apple M2 Max chip, 96 GB unified memory). Under this configuration, a full inference workflow—including input validation, preprocessing (type checking, log-transformation, and median imputation), and model prediction—completed in less than 1 s per case. These results confirm that the model imposes negligible computational burden and can be executed efficiently on standard modern clinical workstations.

**FIGURE 3:**
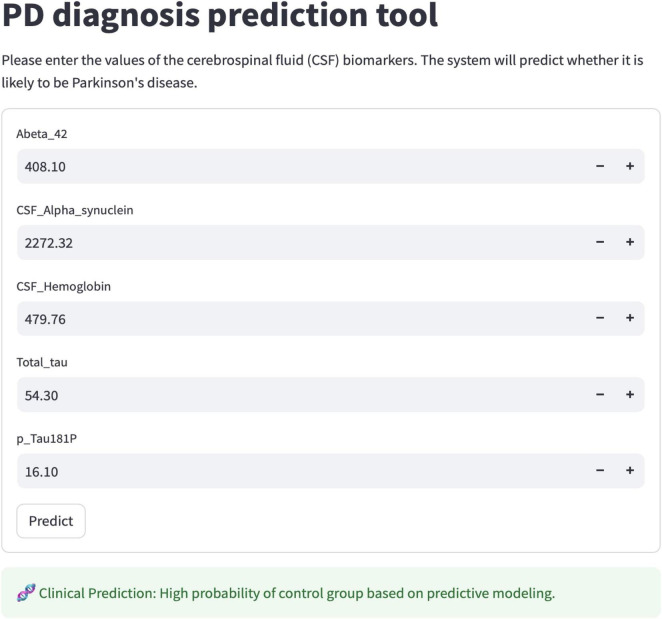
Interactive deployment interface of the random forest prediction model.

The interface accepts numeric CSF biomarker values within physiologically plausible ranges (Aβ42, α-syn, t-tau, p-tau181, hemoglobin). The tool automatically performs input validation, applies the same preprocessing pipeline used during model training, and outputs both the predicted PD probability and an adjustable threshold-based categorical classification. The fully offline implementation facilitates reproducible, rapid, and privacy-preserving predictions suitable for point-of-care use in diverse clinical settings. A screenshot of the deployed interface is shown in Figure 3, illustrating its feasibility for use in resource-limited clinical environments.

## Results

A reproducible workflow combining statistical analysis and supervised ML was implemented to predict PD status from standardized CSF biomarkers (Figure 1). The PPMI cohort served as the primary training and internal validation dataset, providing sufficient variability for model development. After preprocessing and feature selection, five supervised classifiers—L2-logistic regression, random forest, histogram gradient boosting, SVM-RBF, and multilayer perceptron (MLP)—were benchmarked under stratified cross-validation. Random forest achieved the highest discriminative performance and was subsequently retrained on class-balanced data to obtain the final model, which was encapsulated in a lightweight Streamlit-based local interface. External validation in an independent University of Pennsylvania/PPMI cohort demonstrated consistent predictive performance, supporting the robustness and translational potential of the workflow.

Distributions of demographics characteristic and five selected CSF biomarkers were compared between PD and control subjects (Table 1). There were no significant differences in age (*P* = 0.5198) or sex (*P* = 0.5818) distribution between the PD and control groups. For CSF biomarkers, α-syn, t-tau, and p-tau181 were significantly lower in PD than in controls (*P* = 0.0002, 0.0013, and 0.0008, respectively), with Cliff’s delta values ranging from −0.16 to −0.19, indicating small but directionally consistent effects. In contrast, Aβ42 (*P* = 0.4036) and hemoglobin (*P* = 0.2485) did not differ significantly between groups, suggesting limited stand-alone discriminative value for these markers in this cohort. Overall, single-biomarker group separation was modest.

**TABLE 1:** Comparison of CSF biomarkers between Parkinson’s disease and control groups.

Biomarker	Control_Median_IQR	PD_Median_IQR	*P*-value	Cliffs_Delta
Age	62.1 (55.5–69.0)	62.4 (55.2–69.0)	0.5198	−0.03 (negligible, 95% CI −0.13 to 0.07)
Sex	120 (63.2%)	273 (65.8%)	0.5818	–
Hemoglobin	0.0 (0.0–0.0)	0.0 (0.0–0.0)	0.2485	−0.03 (negligible, 95% CI −0.02 to 0.08)
Abeta42	378.1 (311.3–438.6)	367.9 (307.4–427.5)	0.4036	−0.04 (negligible, 95% CI −0.06 to 0.14)
Alpha synuclein	1975.9 (1480.9–2641.9)	1715.7 (1312.7–2183.7)	0.0002	−0.19 (small, 95% CI 0.09 to 0.28)
Total tau	44.4 (36.0–61.0)	41.1 (32.3–52.6)	0.0013	−0.16 (small, 95% CI 0.07 to 0.26)
P-tau181	14.1 (11.1–21.4)	12.5 (9.4–18.5)	0.0008	−0.17 (small, 95% CI 0.07 to 0.26)

*P*-values calculated by Mann–Whitney U test. Cliff’s Delta indicates effect size (negative values indicate lower levels in PD group). Effect size interpretation: negligible < 0.147, small 0.147–0.33, medium 0.33–0.474, large > 0.474. All biomarkers did not follow a normal distribution in both groups (Shapiro–Wilk test *p* < 0.05), and non-parametric tests (Mann–Whitney U test) were uniformly used to report the significance.

Independent contributions of each biomarker were evaluated using multivariable logistic regression including all five CSF measures (Table 2). Logistic regression models were adjusted for potential confounders, including age and sex. Only α-syn remained a statistically significant predictor of PD status (β = −0.3025, *P* = 0.020), indicating that lower α-syn levels are independently associated with increased PD risk after adjustment for the other biomarkers. Aβ42, t-tau, p-tau181, and hemoglobin did not reach statistical significance in the multivariable model, underscoring that their individual effects are limited when modeled jointly with α-syn.

**TABLE 2:** Multivariate logistic regression analysis of biomarkers for predicting Parkinson’s disease.

Predictors	OR	Std. error	z-statistic	*P*-value	[95% CI]	
					Lower	Upper
Const	0.8039	0.090	8.961	0.000	0.628	0.980
Age	0.2032	0.095	2.139	0.032	0.017	0.389
Sex	0.0515	0.189	0.272	0.785	−0.319	0.422
Hemoglobin	−0.0644	0.083	−0.778	0.436	−0.227	0.098
Abeta42	0.0641	0.095	0.677	0.498	−0.121	0.250
Alpha synuclein	−0.3025	0.130	−2.327	0.020	−0.557	−0.048
Total tau	−0.0872	0.135	−0.647	0.518	−0.351	0.177
P-tau181	−0.1185	0.098	−1.203	0.229	−0.311	0.074

OR, odds ratio represented by the logistic regression coefficient (β). CI, confidence interval. A negative coefficient indicates a decreased odds of PD with increasing biomarker levels. Statistically significant results (*P* < 0.05) are in bold. The model adjusts for all listed biomarkers simultaneously.

**FIGURE 4:**
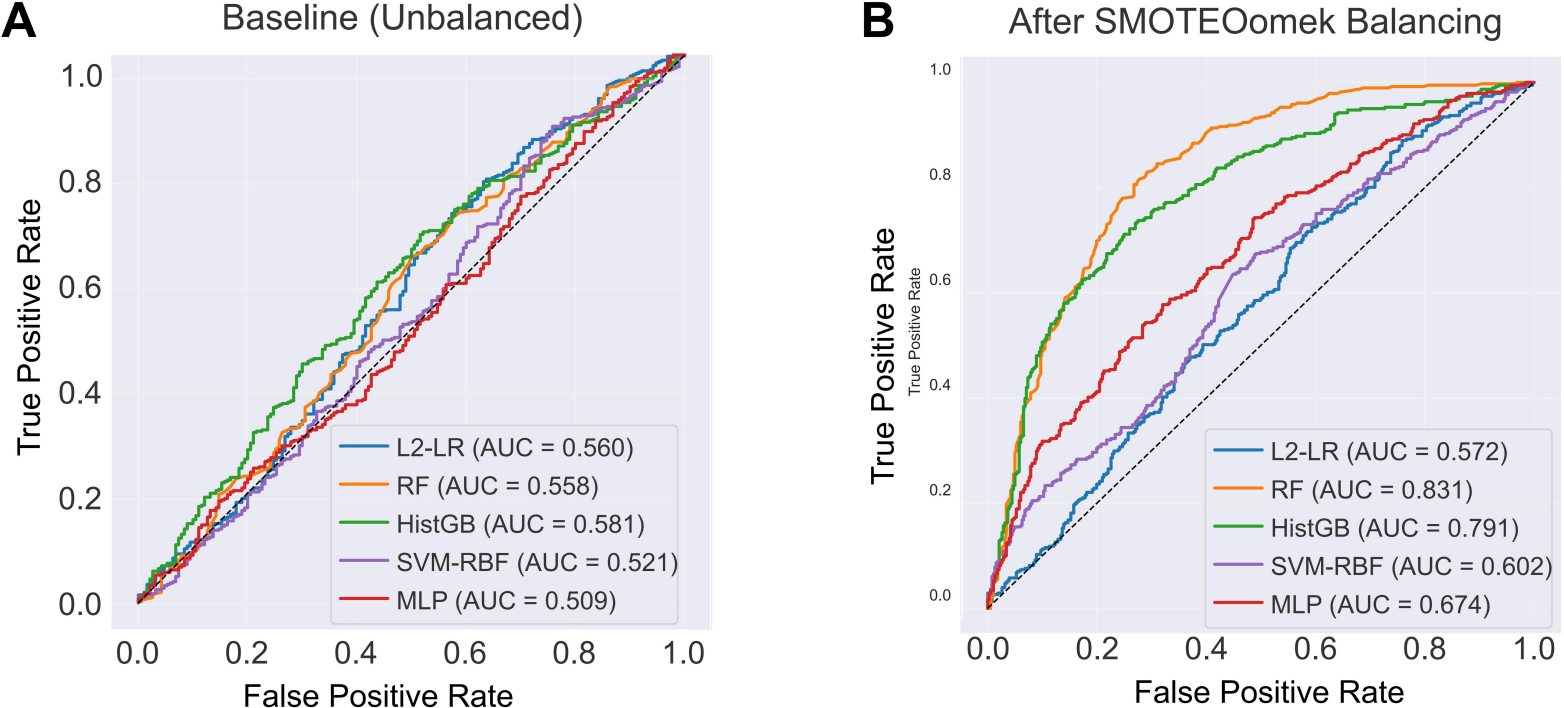
Model performance before and after sample-level balancing. (A) ROC curves of five classifiers trained on the original, unbalanced dataset, showing only modest discriminative performance (AUC range = 0.509–0.581). (B) ROC curves after applying SMOTE-Tomek resampling to balance class representation. RF (AUC = 0.831) and HistGB (AUC = 0.791) showed marked improvement in discrimination, whereas L2-LR, SVM-RBF, and MLP exhibited more moderate gains.

Five supervised classifiers were trained and evaluated using five-fold stratified cross-validation (Figure 4 and Table 3). On the original, imbalanced training data, all algorithms showed modest discriminative ability, with AUC values ranging from 0.509 (MLP) to 0.581 (histogram gradient boosting) (Figure 4A). After application of SMOTE–Tomek resampling within each training fold to balance classes, discrimination improved across all models (Figure 4B). Random forest achieved the highest mean cross-validated AUC (0.831), followed by histogram gradient boosting (0.791), whereas logistic regression and SVM-RBF exhibited intermediate gains. These findings indicate that sample-level class balancing enhances classifier stability and enables better exploitation of nonlinear structure in the CSF biomarker space.

**TABLE 3:** Performance of five machine-learning models before data balancing (unbalanced dataset) and after SMOTETomek data balancing.

	Unbalanced dataset			SMOTETomek data balancing		
Models	AUC (95% CI)	PR–AUC (95% CI)	Brier (95% CI)	AUC (95% CI)	PR–AUC (95% CI)	Brier (95% CI)
L2–Logistic	0.567 ± 0.050	0.722 ± 0.022	0.211 ± 0.008	0.573 ± 0.029	0.557 ± 0.041	0.245 ± 0.005
Random forest	0.558 ± 0.066	0.717 ± 0.044	0.227 ± 0.015	0.828 ± 0.025	0.784 ± 0.036	0.172 ± 0.011
HistGB	0.580 ± 0.039	0.738 ± 0.027	0.251 ± 0.016	0.791 ± 0.031	0.776 ± 0.029	0.191 ± 0.020
SVM–RBF	0.536 ± 0.051	0.709 ± 0.030	0.216 ± 0.005	0.614 ± 0.032	0.635 ± 0.027	0.242 ± 0.005
MLP	0.512 ± 0.037	0.695 ± 0.039	0.305 ± 0.026	0.681 ± 0.026	0.680 ± 0.021	0.229 ± 0.007

Performance metrics of five classifiers trained on the original imbalanced dataset and after achieving class balance via SMOTETomek resampling. All values are expressed as mean ± standard deviation. AUC, area under the receiver operating characteristic curve; PR–AUC, area under the precision-recall curve; Brier, Brier score (lower values indicate superior model reliability). Bold values indicate the best (most favorable) performance for each metric across the five models.

**FIGURE 5:**
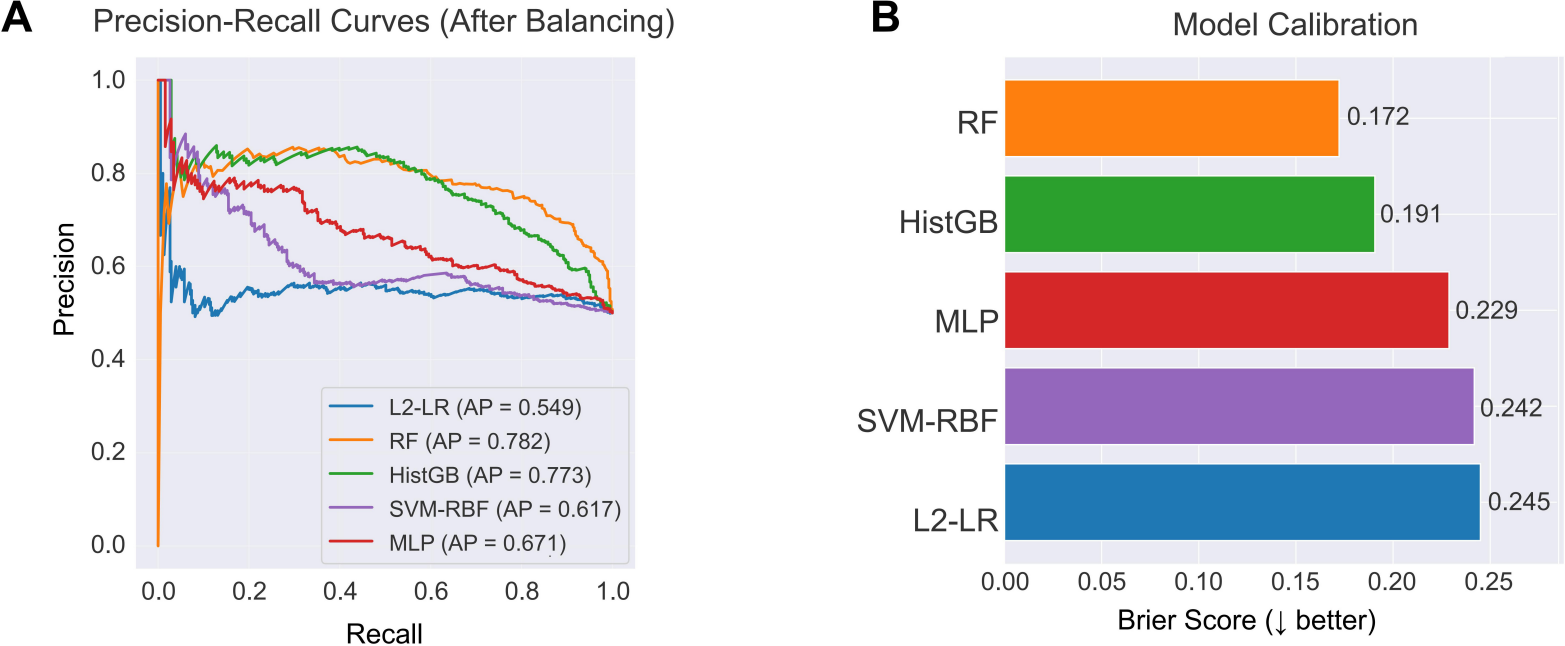
Model discrimination and calibration after class balancing. (A) PR curves of five classifiers trained on the balanced dataset. RF (AP = 0.782) and HistGB (AP = 0.773) achieved the highest discriminative performance, followed by MLP (AP = 0.671), SVM-RBF (AP = 0.617), and L2-LR (AP = 0.549). (B) Calibration performance measured by the Brier score. RF showed the best calibration (Brier = 0.172), followed by HistGB (Brier = 0.191), indicating more reliable probability estimates across the resampled training data.

Class balancing also improved precision–recall behavior and probability calibration (Figure 5). In precision–recall analysis (Figure 5A), random forest and histogram gradient boosting maintained higher precision across a broad range of recall, with average precision scores of 0.782 and 0.773, respectively, reflecting sensitive PD detection with relatively few false positives. Calibration assessment (Figure 5B) showed that the ensemble models yielded the most reliable probability estimates; random forest achieved the lowest Brier score (0.172). Logistic regression and SVM-RBF displayed higher calibration error, indicating less consistent probability scaling. Together, these metrics support the superior reliability of ensemble learners after class balancing.

**FIGURE 6:**
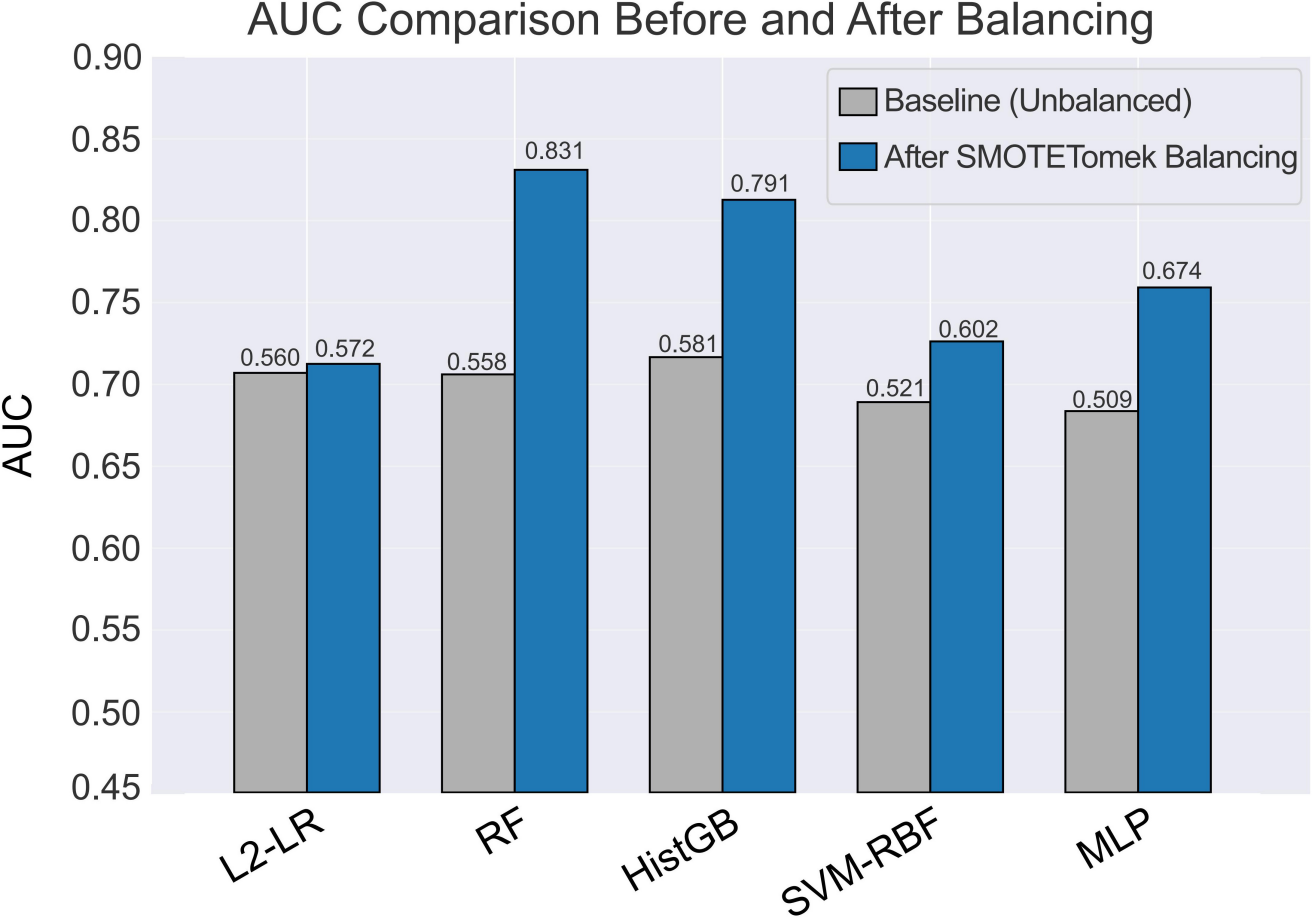
Comparison of classifier AUCs before and after class balancing. Bars show the cross-validated AUC for each algorithm—L2-LR, RF, HistGB, SVM-RBF, and MLP—trained on the original unbalanced dataset (grey) and on the SMOTE-Tomek-balanced dataset (blue).

Changes in model discrimination before and after SMOTE-Tomek class balancing are summarized in Figure 6. On the original imbalanced dataset, AUCs were modest, ranging from 0.509 for the MLP to 0.581 for HistGB. After SMOTE-Tomek resampling, AUCs increased for all five classifiers. Random forest showed the largest gain, with AUC rising from 0.558 to 0.831, followed by histogram-based gradient boosting (0.581 to 0.791). Logistic regression, SVM-RBF, and MLP also improved (0.560 to 0.572, 0.521 to 0.602, and 0.509 to 0.674, respectively). These findings indicate that sample-level class balancing markedly enhances the ability of all models to discriminate PD cases from controls.

**FIGURE 7:**
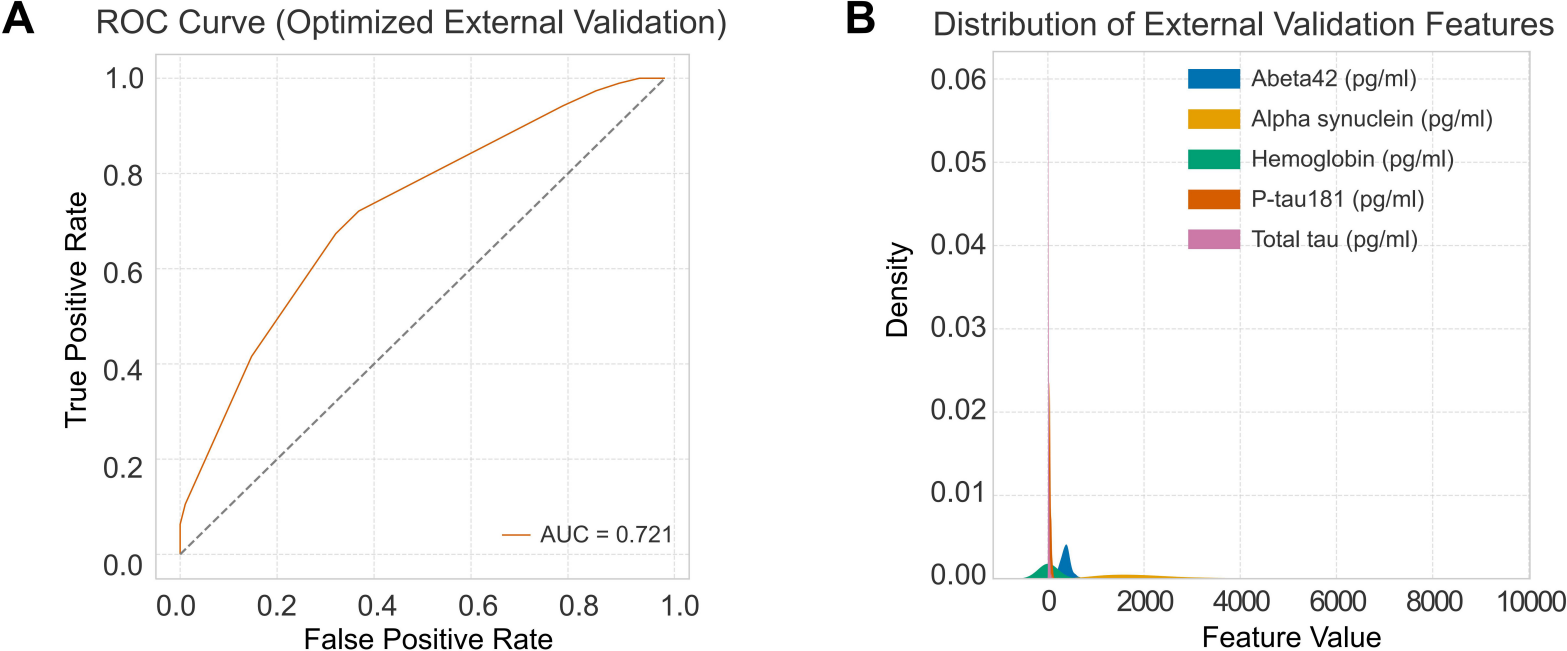
External validation performance and feature distribution. (A) ROC curve of the optimized RF model in the external validation dataset. The model achieved an AUC of 0.721, demonstrating moderate discriminative performance when applied to the independent PPMI CSF cohort. (B) Kernel density estimation of the external validation features, showing the distribution of the five key biomarkers—Aβ42, α-syn, hemoglobin, t-tau, and p-tau181—across PD and control samples.

In the independent PPMI/UPenn cohort, the isotonic-calibrated random forest maintained good generalization performance, with an AUC of 0.721 and a PR-AUC of 0.737 (Figure 7A). Distributions of the five CSF biomarkers (Aβ42, α-syn, t-tau, p-tau181, and hemoglobin) showed comparable ranges and variances between AMP-PD and PPMI (Figure 7B), indicating limited feature drift across cohorts and supporting robustness across assay platforms. These results confirm external validity in a multi-cohort setting and underscore the translational potential of the proposed approach.

The best-performing random forest model was packaged into a lightweight offline desktop application (Figure 3). The interface accepts quantitative CSF biomarker inputs, applies consistent preprocessing (type checking and median imputation), and returns individualized PD risk probabilities with an adjustable decision threshold. The tool runs entirely on local hardware without cloud dependencies, enabling rapid, reproducible risk assessment in resource-constrained clinical environments.

## Discussion and conclusion

In this study, a compact panel of five CSF biomarkers—Aβ42, α-syn, t-tau, p-tau181, and hemoglobin—was used to train a lightweight, locally deployable machine-learning model for first-visit PD prediction. A class-balanced RF was chosen after benchmarking against L2-LR, HistGB, support vector machines, and multilayer perceptrons, as it provided the most favorable trade-off between discrimination, precision–recall performance, and probability calibration. Trained on the PPMI cohort and externally validated in an independent UPenn/PPMI cohort, this routinely measurable CSF panel supported reasonably accurate individualized PD risk estimation.

The final random forest model was packaged as a stand-alone desktop application that performs all input validation, preprocessing, and inference locally, enabling real-time, offline risk estimation from minimal biomarker input on standard hospital hardware without reliance on cloud infrastructure or proprietary software. This design directly addresses two major barriers to clinical adoption of AI tools—limited computational capacity in resource-constrained settings and regulatory constraints on cloud-based processing of sensitive health data—while the availability of a working prototype in a public code repository (e.g., GitHub) promotes transparency, reproducibility, and iterative refinement. To further strengthen reproducibility, the accompanying GitHub repository provides example data structures, complete preprocessing and modeling scripts, and a requirements.txt file documenting the software environment, ensuring that the full analytical workflow can be executed consistently across different systems.

Among the evaluated biomarkers, CSF α-syn emerged as the most robust independent predictor of PD status in multivariable analysis. This aligns with the central pathological role of misfolded α-syn aggregates in nigrostriatal neurons and within Lewy bodies and Lewy neurites (Berman and Siderowf, 2025; Kwon et al., 2022; Leak et al., 2024). Although considerable assay- and cohort-related variability in CSF α-syn measurements has been reported (Berman and Siderowf, 2025; Leak et al., 2024; Magalhães and Lashuel, 2022), the present findings reinforce its diagnostic relevance when considered alongside other neurodegeneration-related markers (Dasari and Medapati, 2025; Parnetti et al., 2019; Zhang et al., 2025). A modest yet reproducible reduction of CSF total tau and p-tau181 in PD, as reported in large cohort and meta-analytic studies, is more consistent with the absence of widespread Alzheimer-type tangle pathology and a distinct, predominantly synuclein-driven mode of neurodegeneration than with a primary tauopathy. Rather than indexing classic AD-like tau deposition, lower tau levels in PD are thought to reflect slower and more selective neuronal injury, altered tau release or clearance, and possible sequestration or alternative processing of tau in the context of α-synuclein pathology (Xiang et al., 2022). Aβ42 and hemoglobin contributed less at the single-biomarker level, but their inclusion in the multivariate panel nonetheless helped stabilize model performance.

From a modeling standpoint, class-balanced ensemble methods, particularly random forests, provided the most favorable balance between discrimination, precision–recall behavior, and probability calibration. Age and sex were not included as predictors, because cases and controls were frequency-matched on these variables and showed no significant between-group differences, making them unlikely to add independent discriminative signal beyond the CSF panel. The modeling objective was to isolate the diagnostic value of a minimal CSF biomarker signature, independent of routine demographic variables, to avoid encoding cohort-specific recruitment patterns and to support generalization to populations with different age–sex structures. Cross-validated performance in AMP-PD and generalization to the independent PPMI cohort indicate that even a small, routinely measurable CSF panel can support reasonably accurate case–control separation. The modest difference between the internal cross-validation AUC (0.831), the held-out test AUC (0.662), and the external validation AUC (0.721) likely reflects cohort heterogeneity, differences in assay platforms, and variations in pre-analytical handling across datasets, which are common challenges in cross-cohort CSF biomarker studies. At the same time, the observed AUCs underscore that CSF biomarkers alone are unlikely to deliver near-perfect classification. This is consistent with prior work and highlights the need to integrate CSF-based models with complementary modalities—such as structural and molecular neuroimaging, genetic risk scores, and digital phenotyping—to capture the full multidimensional heterogeneity of PD and its prodromal states (Gerraty et al., 2023; Lakmala et al., 2022; Luo et al., 2025; Tabashum et al., 2024).

Several limitations must be acknowledged. First, although model development used a large, well-characterized cohort and validation in an independent dataset demonstrated external applicability, both sources are research-oriented consortia. Real-world performance in unselected clinical populations remains to be established (Beaulieu-Jones et al., 2024; Tanaka, 2025). Prospective, multi-center validation in more diverse demographic and clinical settings will be essential to confirm robustness and calibrate decision thresholds. Second, lumbar puncture is inherently invasive and resource-intensive. This constrains the feasibility of CSF-based tools for routine screening or large-scale implementation (Jiao et al., 2024). Adapting this framework to less invasive modalities—such as blood-based biomarkers that increasingly mirror CSF changes or passively acquired digital markers from wearable devices and smartphones—therefore represents an important direction for future work (Rábano-Suárez et al., 2025; Sun et al., 2024). Third, although standard feature-importance and performance metrics were examined, more user-facing interpretability was not fully implemented in the current prototype. Examples include interactive SHAP or LIME visualizations integrated into the application; incorporating such tools could further enhance transparency and clinical trust in model outputs (Hur et al., 2025; Noor et al., 2025). Future work will prioritize multi-center and longitudinal validation across independent cohorts. This will help assess temporal stability, detect potential biomarker drift, and confirm generalizability across diverse clinical and demographic settings.

In summary, this study shows that a small set of routinely measured CSF biomarkers can support a reproducible, locally deployable machine-learning model for PD risk prediction. Coupling curated biofluid data with an offline implementation provides a practical route for biomarker-based decision support in neurodegenerative disease. Future work should validate this framework in multi-center, demographically diverse cohorts. It should also extend the approach to alternative biomarker sources to improve generalizability and facilitate the integration of precision diagnostics into routine neurological care.

## Data Availability

The datasets presented in this study can be found in online repositories. The names of the repository/repositories and accession number(s) can be found below: https://ida.loni.usc.edu/. The cerebrospinal fluid (CSF) biomarker data used in this study were obtained from the Accelerating Medicines Partnership-Parkinson’s disease (AMP-PD) consortium under approved data access agreements. Access to the AMP-PD dataset requires registration and compliance with the consortium’s data use policies. All source code used for data preprocessing, model training, performance evaluation, and local deployment interface development is publicly available at the following GitHub repository: https://github.com/XinchaoHu9966/PDcode.
