# Supplementary material for: A lightweight cerebrospinal fluid biomarker-based model for first-diagnosis prediction of Parkinson’s disease: model development, external validation, and local deployment
Source: Front Aging Neurosci. 2025 Dec 16;17:1723169. doi: 10.3389/fnagi.2025.1723169 (PMC12748221; doi:10.3389/fnagi.2025.1723169)
Supplement: Supplementary file 1 [file Data_Sheet_1.docx]

Table S1. Summary of Data Preprocessing Procedures Applied Before Statistical and Machine-Learning Analyses.

Preprocessing step	Description	Variables
Missing value handling	Median imputation performed within each diagnostic group.	All CSF biomarkers
Outlier handling	Extreme values (>1.5× IQR) were winsorized to the nearest non-outlier boundary to reduce the influence of measurement artifacts.	α-syn, t tau, p-tau181, Aβ
Distribution correction	Natural log-transformation applied to correct right-skewed distributions.	α-synuclein, total tau, p-tau
Normalization	Z-score normalization performed after log-transformation to harmonize biomarker scales.	All continuous biomarke
Leakage prevention	StandardScaler and SMOTE–Tomek were applied only within each training fold during cross-validation to prevent information leakage.	All machine-learning predi
Resampling (training only)	SMOTE–Tomek used to balance classes within each training fold; the test set remained untouched.	Training data
